# Neutrophil-derived apoptotic body membranes-fused exosomes targeting treatment for myocardial infarction

**DOI:** 10.1093/rb/rbae145

**Published:** 2024-12-14

**Authors:** Jingjing Wang, Jingjing Li, Gang Su, Youbin Zhang, Zhu Wang, Yujuan Jia, Qian Yu, Zhenya Shen, Yanxia Zhang, Yunsheng Yu

**Affiliations:** Department of Cardiovascular Surgery of the First Affiliated Hospital & Institute for Cardiovascular Science, Suzhou Medical College of Soochow University, Soochow University, Suzhou 215006, P. R. China; Department of Cardiovascular Surgery of the First Affiliated Hospital & Institute for Cardiovascular Science, Suzhou Medical College of Soochow University, Soochow University, Suzhou 215006, P. R. China; Department of Cardiovascular Surgery of the First Affiliated Hospital & Institute for Cardiovascular Science, Suzhou Medical College of Soochow University, Soochow University, Suzhou 215006, P. R. China; Department of Cardiovascular Surgery of the First Affiliated Hospital & Institute for Cardiovascular Science, Suzhou Medical College of Soochow University, Soochow University, Suzhou 215006, P. R. China; Department of Cardiovascular Surgery of the First Affiliated Hospital & Institute for Cardiovascular Science, Suzhou Medical College of Soochow University, Soochow University, Suzhou 215006, P. R. China; Department of Cardiovascular Surgery of the First Affiliated Hospital & Institute for Cardiovascular Science, Suzhou Medical College of Soochow University, Soochow University, Suzhou 215006, P. R. China; State and Local Joint Engineering Laboratory for Novel Functional Polymeric Materials, College of Chemistry, Chemical Engineering and Materials Science, Soochow University, Suzhou 215123, P. R. China; Department of Cardiovascular Surgery of the First Affiliated Hospital & Institute for Cardiovascular Science, Suzhou Medical College of Soochow University, Soochow University, Suzhou 215006, P. R. China; Department of Cardiovascular Surgery of the First Affiliated Hospital & Institute for Cardiovascular Science, Suzhou Medical College of Soochow University, Soochow University, Suzhou 215006, P. R. China; Department of Cardiovascular Surgery of the First Affiliated Hospital & Institute for Cardiovascular Science, Suzhou Medical College of Soochow University, Soochow University, Suzhou 215006, P. R. China

**Keywords:** myocardial infarction, neutrophil-derived apoptotic body, exosome, membrane fusion

## Abstract

Myocardial infarction (MI) poses a substantial threat to human health, prompting extensive research into effective treatment modalities. Preclinical studies have demonstrated the therapeutic potential of mesenchymal stem cell-derived exosomes for cardiac repair. Despite their promise, the inherent limitations of natural exosomes, mainly their restricted targeting capabilities, present formidable barriers to clinical transformation. To address this, it is proposed to enhance their targeting specificity and retention in infarcted myocardium by fusing exosomes with neutrophil-derived apoptotic body membranes (NAM). These NAM inherit the surface signals from neutrophils, which allow them to home in on the damaged tissues and participate in regulating inflammatory responses. In this current work, we utilized a membrane fusion technique to create NAM-fused exosomes (NAM-Exo) for MI treatment. Compared to their native counterparts, NAM-Exo demonstrated enhanced adhesion to inflammatory endothelial cells, replicating the neutrophil recruitment mechanism at sites of myocardial injury in MI. Furthermore, our findings revealed that NAM-Exo not only significantly modulated inflammation responses but also promoted angiogenesis in a mouse model of MI, ultimately leading to improved cardiac function and ventricular remodeling post-treatment. These results underscore the potential of membrane fusion as an effective strategy to enhance the therapeutic efficacy of exosome-based cardiac repair and regeneration therapies, thereby paving the way for their translation into clinical practice.

## Graphical abstract

**Figure:**
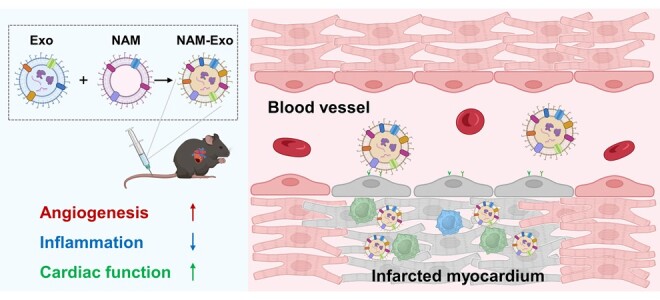

## Introduction

Myocardial infarction (MI) exerts a significant impact on individuals’ well-being and longevity owing to its substantial morbidity and mortality [1]. Current clinical treatments for MI include pharmacologic therapy, bypass operation and percutaneous coronary intervention [2, 3]. While these interventions can marginally ameliorate patient symptoms, they may fail to repair infarcted myocardium due to the limited regenerative capacity of adult mammalian cardiomyocytes (<1%) [4–6]. Therefore, there is a pressing need for novel approaches to address heart infarction. Cell therapy has emerged as a promising avenue for promoting myocardial repair and enhancing cardiac function by transplanting cells, such as mesenchymal stem cells (MSCs) [7–9], cardiomyocytes derived from induced pluripotent stem cells, or human embryonic stem cells [10, 11] into the infarcted area. However, cell therapy is encumbered by challenges such as low storage and shipping stability of live cells, poor retention in the heart and adverse effects, including tumorigenicity, arrhythmogenicity and immune rejection [12].

The mechanistic underpinnings of the functional improvements observed with cell therapy remain poorly understood. However, numerous studies indicate that transplanted cells may exert their effects through paracrine action, mediated by the release of extracellular vesicles (EVs), other bioactive factors or both [13]. Consequently, there is growing interest in cell-free therapies, particularly those centered on EVs, obviating the need to transplant large numbers of cells while yielding a better-defined and less expensive product. Exosomes, a subtype of small EVs ranging in diameter from 40 to 150 nm, harbor diverse biological cargo contents, including lipids, proteins and RNAs, facilitate cell-to-cell communication, and transmit various cellular signals [14–16]. Numerous reports have highlighted the therapeutic potential of exosomes in cardiac protection, encompassing benefits such as enhanced myocardial angiogenesis, reduced oxidative stress, limited inflammatory response, decreased cardiomyocyte death and reduced MI size [17–20]. For example, our previous study showed that MSC-derived exosomes could inhibit cardiac fibrosis and improve cardiac function in MI mice [17]. However, the inherent limitations of exosomes hinder their therapeutic potential, posing a challenge for clinical translation. For instance, natural exosomes cannot target the infarcted myocardium and exhibit a short half-life in peripheral blood circulation; this limitation leads to rapid clearance by the mononuclear-phagocyte system, particularly in the liver, spleen and lung tissues [21]. These factors limit their potential to promote cardiac recovery. A potential solution to overcome these strategies and facilitate the clinical application of exosome therapy involves enhancing their targeting specificity and prolonging their retention in infarcted myocardium while preserving their therapeutic efficacy. In pursuit of this goal, exosomes have been fused with cell membranes, such as those of platelets [22], monocytes [23] and macrophages [24] using physical techniques. This engineering approach enhances the versatility and complexity of the natural cell membranes, preserving all biologically relevant surface components by integrating them with the exosome membrane. These preserved features include properties that are advantageous for immune evasion and targeting, which are crucial for the development of targeted drug delivery systems. Consequently, exosomes that have undergone membrane fusion hold greater potential for clinical research. In addition, exosomes have also been modified with target peptides, including CHP (CSTSMLKAC) [25], CTP (APWHLSSQYSRT) [26, 27], CMP (WLSEAGPVVTVRALRGTGSW) [28] and IMTP (CSTSMLKAC) [29], employing a range of chemical or biological techniques.

Following MI, a significant influx of neutrophils occurs within the injured heart, instigating an inflammatory response [30, 31]. However, the persistent infiltration of neutrophils led to delayed inflammatory resolution, potentially impeding tissue repair and contributing to adverse remodeling and impaired cardiac function. Typically, when neutrophils undergo apoptosis, macrophages in the inflammatory site phagocytize them, triggering a shift in macrophage phenotype and increased secretion of anti-inflammatory factors, thereby promoting tissue homeostasis and resolution of inflammation [32–34]. Simultaneously, apoptotic neutrophils secrete mediators such as annexin A1 and lactoferrin, which inhibit further neutrophil recruitment [35]. Therefore, the delicate balance between neutrophil survival and apoptosis plays a crucial role; however, inducing neutrophil apoptosis *in situ* presents challenges. While the provision of apoptotic neutrophils may aid in promoting cardiac repair, direct engraftment of exogenous apoptotic cells could provoke inflammation due to cell debris or inflammatory products. Apoptotic bodies (ABs) are EVs generated during cell apoptosis, ranging in size from 50 nm to 5 μm. They form through membrane blebbing during the foaming of the plasma membrane of apoptotic cells and are subsequently lysed and released under the mediation of caspase [36]. Neutrophil-derived ABs inherit surface signal molecules from their parent cells, enabling them to target damaged tissues and participate in regulating inflammatory response. Cell membrane biomimetic strategies have recently gained traction in biomedical applications [37–39]. For instance, engineered neutrophil-derived ABs were developed by coating mesoporous silica nanoparticles with the membrane of native neutrophil-derived ABs. These biomimetic ABs mimic the role of apoptotic neutrophils, exhibiting robust binding affinity with inflammatory endothelial cells. Meanwhile, they replicated the immunomodulatory activities of their parent cells through the inheritance of apoptosis-related signaling molecules [40]. Therefore, it is anticipated that exosomes modified with the membrane of native neutrophil-derived ABs could enhance their targeting and retention in the injured heart, thereby improving cardiac repair.

**Figure 1.**
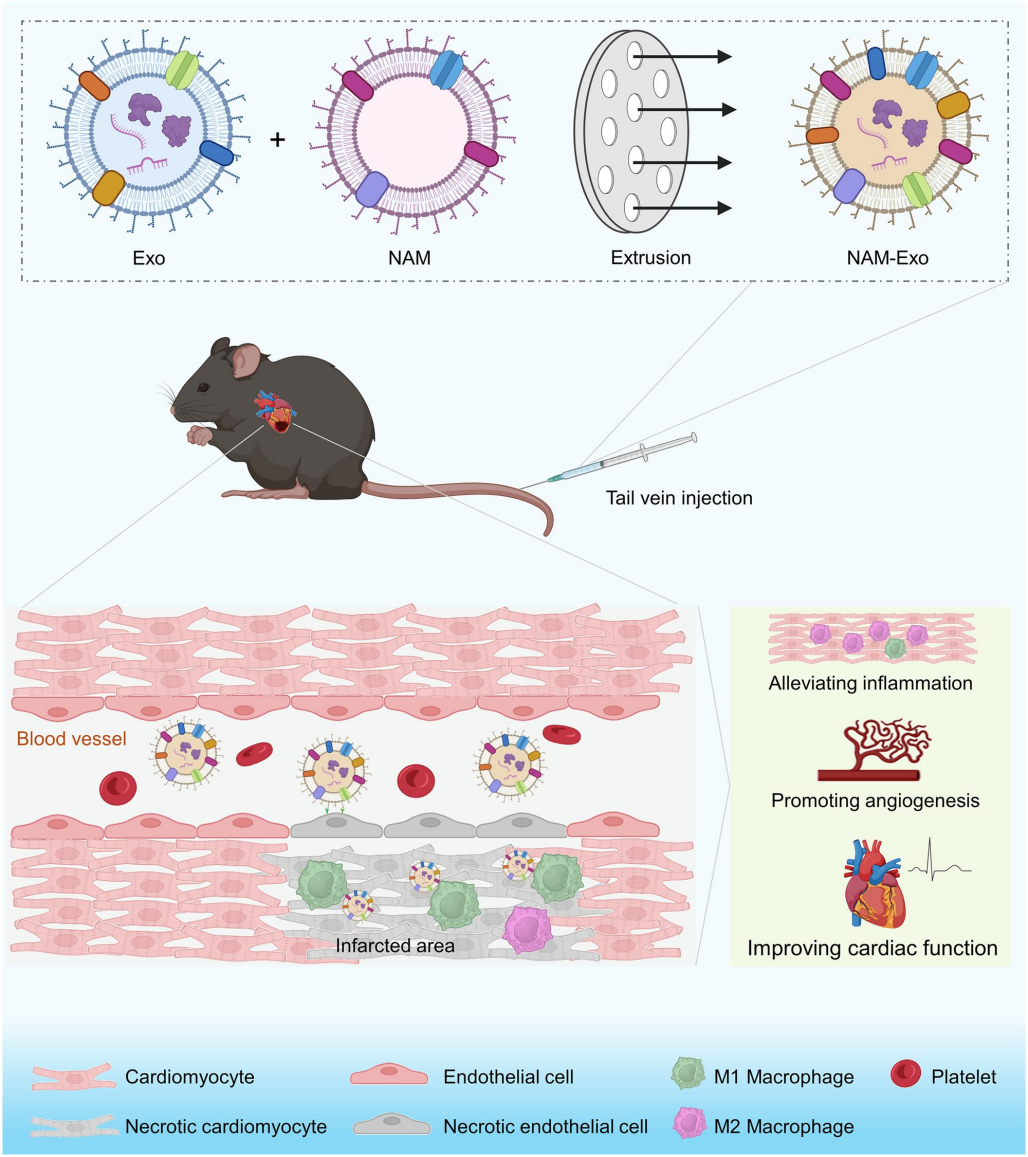
Schematic illustration of neutrophil-derived AB membrane-fused exosomes (NAM-Exo) for treatment of MI. The image was generated using BioRender.com.

In this study, a fusion exosome termed neutrophil-derived AB membranes-fusion exosomes (NAM-Exo) was engineered to mimic the chemotactic behavior of neutrophils toward inflammatory regions, thereby enhancing the exosome’s targeting ability and improving its therapeutic efficacy for treating MI (Figure 1). Neutrophil-derived AB membranes (NAM) were isolated from apoptotic peripheral blood neutrophils, while exosomes were derived from mouse bone marrow-derived MSCs (BMSCs). Subsequently, fused exosomes combining these components were obtained using the ‘co-incubation and extrusion’ method to prepare NAM-Exo. The properties of ABs and NAM-Exo were thoroughly characterized. The inflammation-tropism property of NAM-Exo was assessed using TNF-α-stimulated endothelial cells, and its uptake by various cell types (including cardiomyocytes, fibroblasts and macrophages) was evaluated *in vitro*. Finally, NAM-Exo was administered via tail vein injection to MI mice to assess its targeting ability to infarcted myocardium and its therapeutic efficacy in alleviating inflammation, promoting angiogenesis and improving cardiac function.

## Methods and materials

All animal procedures were conducted with approval from the Ethics Committee of Soochow University (approval No. SUDA20221124A18).

### Collection and characterization of neutrophil-derived ABs

Detailed procedures for preparing apoptotic neutrophils are described in the Supplementary Information. ABs were isolated from the culture media and centrifuged at 50 g for 5 min twice to eliminate cells and debris. The resulting supernatant was centrifuged at 1000 g for 10 min to obtain the ABs pellet. The concentrated ABs were resuspended in phosphate-buffered saline (PBS) and quantified using a bicinchoninic acid (BCA) protein assay kit. Morphological and size analyses of ABs were performed using transmission electron microscopy (TEM) and dynamic light scattering (DLS). Fluorescence staining and flow cytometry analysis of ABs were conducted using an Annexin V-fluorescein isothiocyanate (FITC) apoptosis assay kit (Beyotime, China). Western blotting was performed to characterize the protein constitution of the ABs.

### Fusion of neutrophil-derived apoptotic body membranes with exosomes

Detailed procedures for isolating and characterizing BMSC and BMSC-derived exosomes are described in the Supplementary Information and Figure S1. NAM was obtained following previously reported protocols [40]. Briefly, the ABs were resuspended in 0.25× PBS at 4°C for 2 h, followed by sonication for 5 s. After double washing with distilled water twice, the membrane was concentrated by centrifugation at 5000 g for 10 min. The membrane suspensions were subjected to at least 10-circle extrusion through 200 nm polycarbonate membranes to obtain the final NAM. Subsequently, a mixture of NAM and Exo, at the protein ratios of 1:1, was incubated at 37°C for 15 min and extruded gently 10 times with a 200-nm polycarbonate membrane (Cytiva, USA) to generate NAM-Exo.

The morphology and size of NAM-Exo were assessed using TEM and DLS analysis. To confirm the successful membrane fusion, NAM was stained with DiO at 37°C for 30 min and then centrifuged at 5000 g for 15 min to remove excess dye. Similarly, Exo was stained with DiD at 37°C for 30 min and then centrifuged at 110 000 g for 70 min. Subsequently, NAM-Exo was prepared with DiO-labeled NAM and DiD-labeled Exo following the NAM-Exo preparation protocol. NAM-Exo fusion was observed using a fluorescence microscope (Nikon, Japan). Furthermore, sodium dodecyl sulfate (SDS) polyacrylamide gel electrophoresis was employed to confirm the presence of neutrophil AB membrane proteins on the surface of NAM-Exo.

### Targeting ability of NAM-Exo *in vitro* and *ex vivo*

Human umbilical vein endothelial cells (HUVECs, 1×$10^{6}$) were seeded in confocal dishes with tumor necrotic factor (TNF)-α (100 ng/ml) in Dulbecco’s modified eagle medium (DMEM) (low glucose) for 6 h. Subsequently, cells were washed and incubated with 50 μg/ml DiI-labeled Exo and NAM-Exo at 4°C for 15 min. Following incubation, cells were washed, fixed with 4% paraformaldehyde (PFA) and subjected to immunofluorescence (IF) staining for the adhesive protein intercellular adhesion molecule-1 (ICAM-1) using a primary anti-ICAM-1 antibody (Abcam, UK) and an Alexa Fluor 488-secondary antibody. Meanwhile, to assess and compare the uptake efficiency of NAM-Exo by different cell types, DiI-labeled NAM-Exo was incubated with cardiomyocytes (HL-1), fibroblasts (NIH3T3) and macrophages (RAW 264.7) for 24 h.

The detailed procedures for establishing the MI mouse model are outlined in the Supplementary Information. Mice were divided into three groups, MI, Exo and NAM-Exo, to evaluate the biological distribution *in vivo*. DiR-labeled Exo and NAM-Exo (100 μg) were administered via tail vein injection following MI induction. DiR-labeled Exo or NAM-Exo distribution was monitored in real-time over 24 h using an *in vivo* optical imaging system (PerkinElmer, Germany) with excitation/emission wavelengths of 754/778 nm. DiR fluorescence distribution in the heart, brain, liver, spleen, lung and kidney was further imaged by *in vivo* imaging system.

### *In-vivo* analysis of inflammation response

To determine the inflammatory response in the infarcted heart, MI mice were randomly assigned to three groups: MI, Exo and NAM-Exo. Heart tissue was collected at 7 days post-treatment. The inflammatory response in the left ventricular anterior wall was evaluated by quantitative PCR (qPCR). Total RNA was extracted from mouse heart tissues using ${\mathrm{TRIzol}}^{\mathrm{TM}}$ (Thermo Fisher Scientific, USA), followed by reverse transcription to complementary DNA (cDNA) using the PrimeScript RT Master Mix Kit (Takara Bio, Japan). The mRNA levels were analyzed by qPCR using TB Green^®^  *Premix Ex Taq*™ II (Takara) on a Roche LightCycler480 PCR System (Roche Diagnostics, Austria). The relative expression levels of genes were calculated using the $2^{-\Delta \Delta \mathrm{Ct}}\;$method. Glyceraldehyde 3-phosphate dehydrogenase (GAPDH) was the internal control; the primer sequences are detailed in Table S1.

Tissue sections were obtained with hematoxylin and eosin staining (H&E, Solarbio, China) to assess inflammation cell infiltration, cell nuclear arrangement and cardiomyocyte muscle fiber integrity. Meanwhile, immunofluorescence staining was employed to analyze the expression levels of inducible nitric oxide synthase (iNOS, an M1 macrophage marker) and CD206 (an M2 macrophage indicator) in these tissue sections.

### *In vivo* evaluation of angiogenesis, cardiac fibrosis and heart function

The mice were randomly divided into four groups: Sham, MI, Exo and NAM-Exo. The Sham group underwent only the Sham operation without any treatment. PBS (100 μl), Exo (100 μg) and NAM-Exo (100 μg) were administered via the tail vein of mice 1 day after MI, and subsequently injected once a week for 4 weeks.

Four weeks postoperatively, mouse hearts were harvested, paraffin-embedded and sectioned into 7-μm slices. Masson’s trichrome staining (Solarbio) was performed following the manufacturer’s instructions to assess cardiac fibrosis. Furthermore, heart weight to body weight ratio (HW/BW) and heart weight to tibial length ratio (HW/TL) were measured to examine ventricular remodeling post-MI. CD31 immunohistochemical staining (Abcam, UK) was performed to evaluate capillary density in heart tissues. Images were photographed using a NanoZoomer S210 (Hamamatsu Photonics, Japan) and analyzed with ImageJ software.

Cardiac function in mice, including left ventricular ejection fraction (LVEF) and fraction shortening (LVFS), was assessed using a Vevo 2100 echocardiography imaging system (VisualSonics, Canada) at baseline, 7, 14 and 28 days post-surgical intervention for MI.

### Biosafety evaluation of NAM-Exo therapy

MI mice were randomly assigned to MI, Exo and NAM-Exo groups to assess NAM-Exo biosafety in mice. At 28 days postoperatively, mice were euthanized, and serum and major organs were collected. Peripheral blood was obtained via eye blood sampling, centrifuged at 3000 rpm for 10 min to obtain serum, and analyzed for serum alanine transaminase (ALT), aspartate transaminase (AST) and blood urea nitrogen (BUN) using commercial kits (Solarbio). Histopathological changes in major organs (liver, spleen, lung and kidney) were observed using H&E staining.

## Results and discussion

### Preparation and characterization of ABs and NAM-Exo

**Figure 2.**
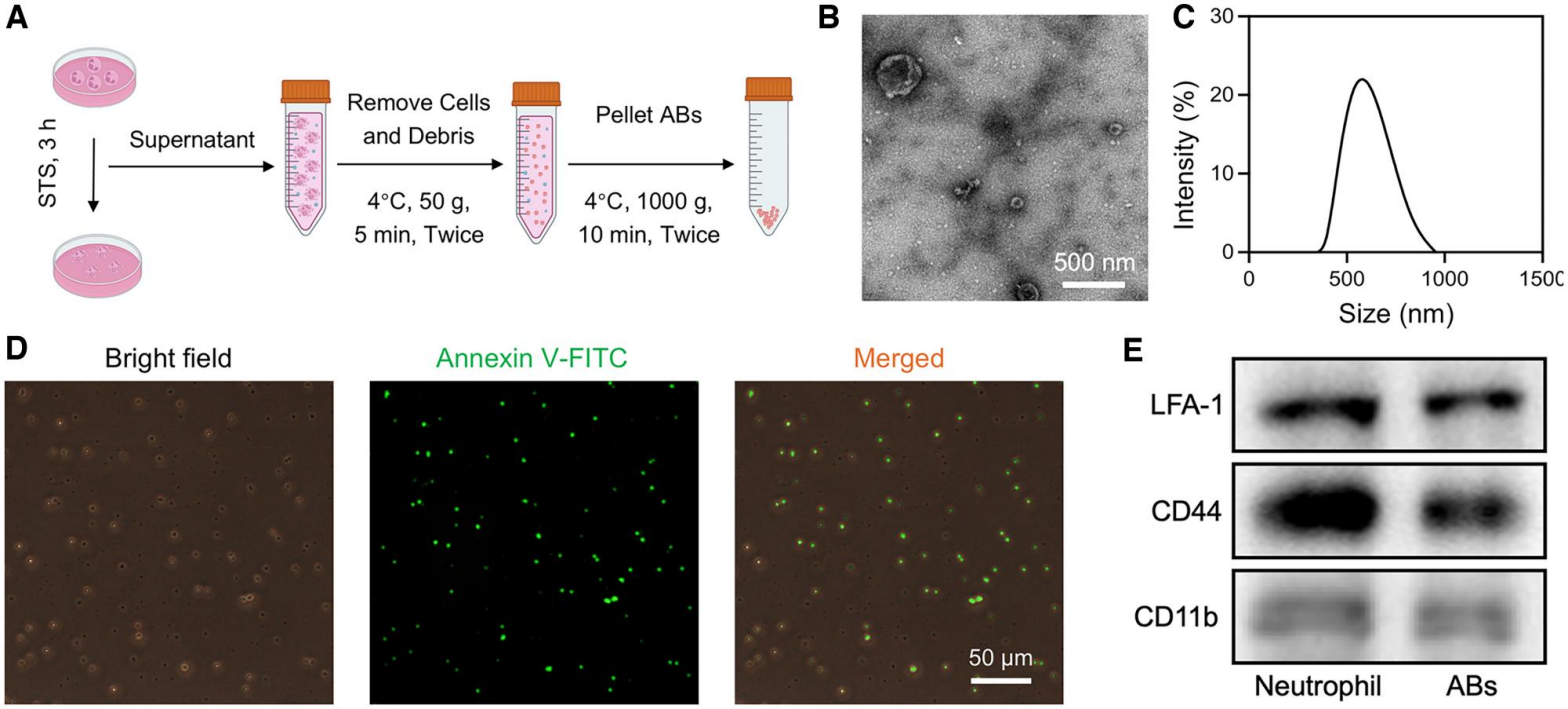
Preparation and characterization of neutrophil-derived ABs. (**A**) Schematic representation depicting the generation of ABs through induction of neutrophil apoptosis followed by serial centrifugation steps. (**B**) Representative TEM image of ABs. (**C**) Size distribution of ABs. (**D**) Annexin V staining of ABs. (**E**) Western blot analysis of membrane-specific protein markers of neutrophils and ABs.

Neutrophil-derived ABs were isolated from drug-induced apoptotic neutrophils by gradient centrifugation (Figure 2A) [40]. Results from TEM and DLS revealed that ABs exhibited vesicle-like structures with a diameter of 350–1000 nm (Figure 2B and C), consistent with previous studies [36]. During cell apoptosis, phosphatidylserine on the inner side of the cell membrane becomes exposed on the outer side, increasing phosphatidylserine on the apoptotic cell [41, 42]. Annexin V, a commonly used probe for molecular imaging of apoptotic cells, is a member of the Annexin family of phospholipid-binding proteins. It binds with high affinity to negatively charged phosphatidylserine (PS) in Ca^2+^. Results from Annexin V fluorescence staining (Figure 2D) and flow cytometry (Figure S2) indicated a high level of phosphatidylserine expression on ABs.

After MI, a substantial influx of neutrophils occurs in the infarcted myocardium, facilitated by specific membrane proteins involved in cell migration, adhesion and homing, such as lymphocyte function-associated antigen-1 (LFA-1), CD44 and CD11b [30, 31]. This study investigated the surface inflammatory adhesion-associated protein markers of ABs through western blotting. The results revealed that typical molecules on neutrophils were inherited by ABs (Figure 2E), suggesting that ABs may possess the potential to target the inflammatory endothelium similar to natural neutrophils.

**Figure 3.**
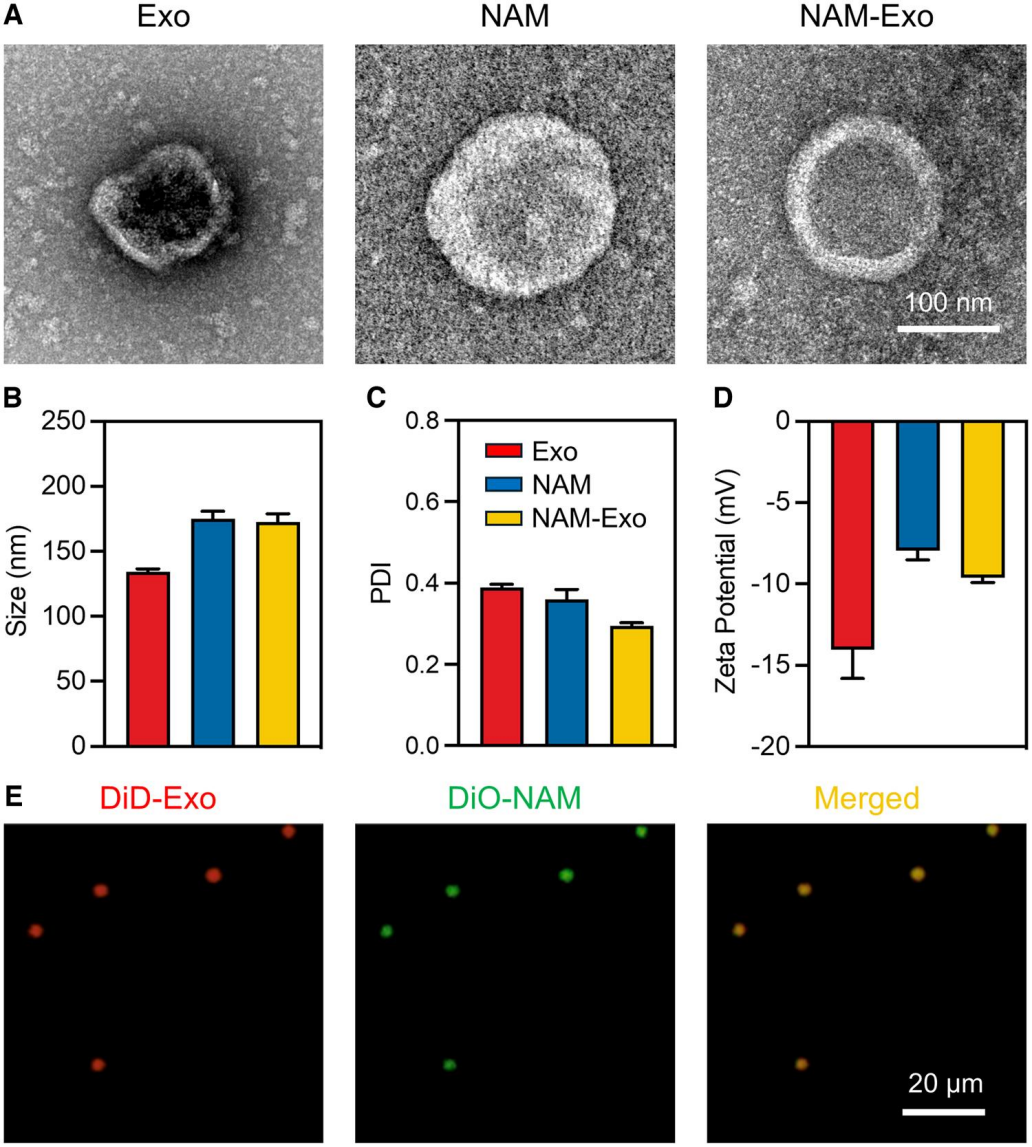
Generation and characterization of neutrophil-derived AB membranes-fused exosomes (NAM-Exo). (**A**) Representative TEM images showcasing Exo, NAM, and NAM-Exo. (**B**) Size, (**C**) polydispersity index (PDI), and (**D**) zeta potential of Exo, NAM and NAM-Exo. (**E**) Fluorescence microscopy images capturing NAM-Exo formed by merging DiD-labeled Exo and DiO-labeled NAM.

NAM was isolated by removing the vesicle components of ABs through hypotonic treatment and sonication. Exosomes derived from BMSCs were isolated from a conditioned culture medium via ultracentrifugation, and the characteristics of both BMSCs and exosomes are demonstrated in Figure S3. Subsequently, NAM was fused with exosomes through 200 nm membranes for at least 10 cycles to generate NAM-Exo. As shown in Figure 3A, NAM-Exo retained a round shape after fusion. NAM-Exo exhibited a diameter of 172.83 ± 6.10 nm, comparable to that of NAM (175.10 ± 5.53 nm) and larger than that of exosomes (134.37 ± 2.20 nm, Figure 3B); the polydispersity index (PDI) of NAM-Exo was lower than that of NAM and exosomes (Figure 3C). The zeta potential measurements of the three nanoparticles all revealed negative charges, with the order being exosomes < NAM-Exo < NAM (Figure 3D), indicating a modification in the membrane composition of NAM-Exo compared to exosomes. These negative charges potentially decrease nonspecific cellular uptake and prolong circulation by electrostatic repulsion with negatively charged cell membrane surfaces [43].

To validate the successful fusion between NAM and Exo, membrane fusion studies were conducted using fluorescence colocalization and protein analysis. DiD-labeled Exo (red color) was initially combined with DiO-labeled NAM (green color) and subsequently assessed. Immunofluorescent staining revealed colocalization of DiD-labeled Exo and DiO-labeled NAM, resulting in merged yellow dots (Figure 3E), indicating successful membrane fusion between Exo and NAM. The protein composition of NAM-Exo was assessed using Coomassie brilliant blue staining, revealing some identical protein expression patterns with both NAM and Exo (Figure S4). These results collectively suggest that Exo and NAM can be efficiently fused through extrusion to produce NAM-Exo, which might simultaneously possess the therapeutic properties of exosomes and the chemotactic abilities of neutrophils.

### Targeting ability of NAM-Exo *in vitro* and *ex vivo*

**Figure 4.**
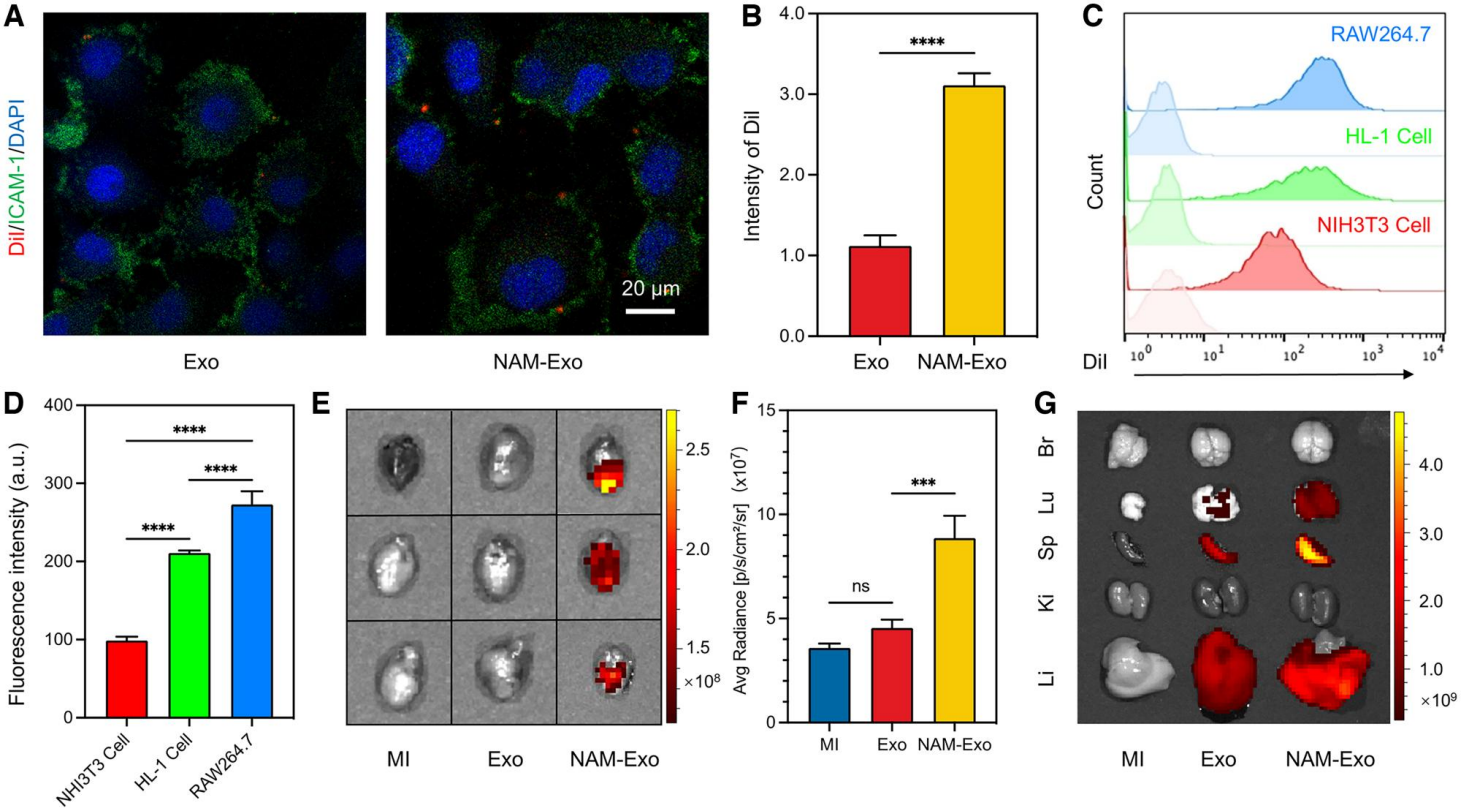
Targeting ability of NAM-Exo *in vitro* and *ex vivo*. (**A**) Representative fluorescence microscopic images demonstrate the adherence of NAM-Exo to the inflamed endothelium *in vitro*. (**B**) Quantitative assessment of fluorescence intensity of DiI-labeled Exo/NAM-Exo. (**C** and **D**) Flow cytometry analysis and quantification of mean fluorescence intensity of DiI-labelled NAM-Exo co-incubated with NHI3T3 Cell, HL-1 Cell and RAW264.7. (**E** and **F**) Fluorescence imaging and qualification of *ex vivo* hearts from MI mice at 24 h after injection with DiR-labeled Exo and NAM-Exo. (**G**) *Ex vivo* images of other major organs (brain, Br; lung, Lu; spleen, Sp; kidney, Ki; liver, Li). Data are mean ± SD (*n *=* *3; ***P <* *0.001, ****P <* *0.0001, ns stands for no significant difference).

Following MI, neutrophils swiftly migrate to the infarcted myocardium, a process heavily reliant on their membrane proteins LFA-1, CD44 and CD11b, crucial for recognizing inflammatory endothelium [41]. These interactions involve various mechanisms, such as LFA-1’s interaction with ICAM-1, which is significantly upregulated by endothelial cells post-MI [44, 45]. Since NAM inherits surface signal molecules from neutrophils, demonstrating remarkable inflammation tropism [40]. Therefore, to investigate whether NAM-Exo could target inflammatory endothelium *in vitro*, TNF-α treated endothelial cells were employed to simulate inflammatory endothelium and were cocultured with DiI-labeled Exo or NAM-Exo. More significant red fluorescence (from DiI) was observed in endothelial cells cocultured with NAM-Exo compared to Exo alone (Figure 4A and B), suggesting enhanced inflammatory tendencies of exosomes facilitated by NAM-mediated adhesion interactions. Therefore, it is conceivable that the adhesive properties of NAM-Exo to inflammatory endothelium correlate with the presence of adhesion molecules (such as LFA-1, CD44 and CD11b) on the neutrophil cell membrane. This suggests that the membrane fusion technique preserves the inherent targeting ability of the original cells, which is consistent with previous reports [46, 47].

Moreover, upon binding to inflammatory endothelium, NAM-Exo further infiltrates the infarct tissue within the infarcted myocardium and is subsequently internalized by various cell types. The uptake ability of different cells for NAM-Exo was investigated. DiI-labeled NAM-Exo was cocultured with cardiomyocytes, fibroblasts and macrophages, each starting with the same initial cell density. Flow cytometry showed that macrophages significantly absorbed more DiI-labeled NAM-Exo than fibroblasts and cardiomyocytes (Figure 4C and D). This phenomenon may be attributed to the elevated phosphatidylserine expression on the NAM-Exo. The recognition and phagocytosis of apoptotic cells by macrophages are likely due to increased phosphatidylserine exposure on apoptotic membranes, which specifically binds to phosphatidylserine receptors in macrophages [33].

A targeting strategy aimed at augmenting the retention of exosomes could enhance their therapeutic effectiveness. Following MI induction, DIR-labeled NAM-Exo or Exo were administered via tail vein injection. Twenty-four hours post-injection, major organs, including heart, brain (Br), lung (Lu), spleen (Sp), kidney (Ki) and liver (Li), were obtained and examined by an *in vivo* (IVIS) spectrum imaging system. The results revealed that NAM-Exo could target the MI heart with better retention than Exo at 24 h after injection (Figure 4E and F). NAM-Exo retention in the MI mice was evaluated using an *in vivo* and *ex vivo* imaging system. As depicted in Figure 4G and Figure S5, we observed the biological distribution of NAM-Exo, which also resides in the liver, spleen and lung, similar to exosomes. However, the number of NAM-Exo significantly surpassed that of Exo, confirming that NAM modification technology effectively improves exosome residence *in vivo*, thereby optimizing their therapeutic impact.

### NAM-Exo reduces inflammation response *in vivo*

Acute myocardial ischemia typically triggers cellular injury and demise across myocardial components (such as cardiomyocytes, endothelial cells and cardiac fibroblasts), initiating an acute pro-inflammatory reaction and releasing various pro-inflammatory factors. These factors further induce the migration and accumulation of inflammatory cells in the infarct zone, exacerbating the inflammatory response [33]. Neutrophil apoptosis, followed by their removal, is a hallmark of inflammation resolution. Apoptotic neutrophils facilitate intercellular communication through the release of ABs. The NAM inherits surface signal molecules from ABs, which could initiate macrophage polarization and contribute to the regulation of inflammatory resolution.

**Figure 5.**
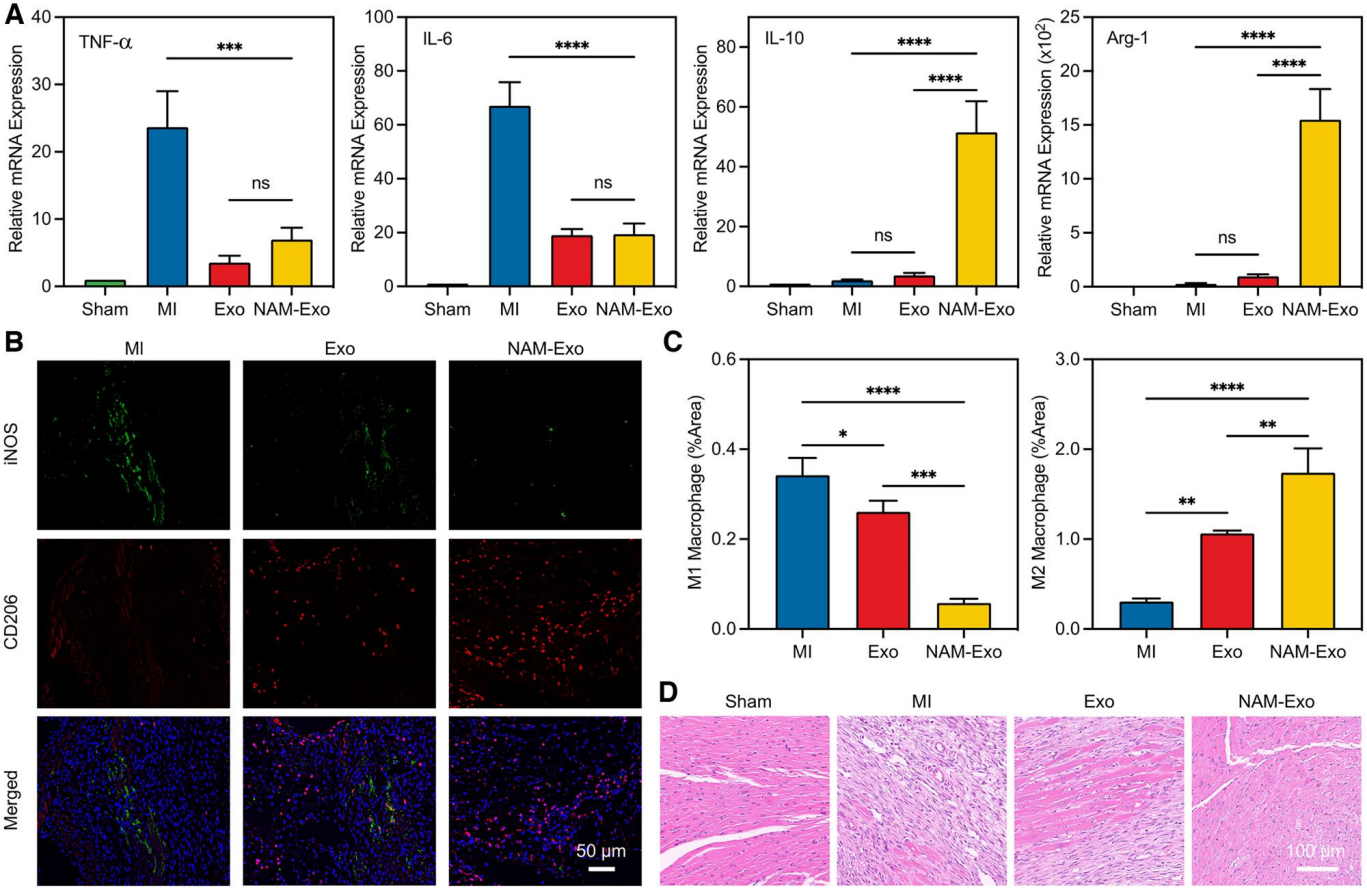
*In vivo* immunomodulatory capacity of NAM-Exo. (**A**) Relative mRNA expression of inflammatory and anti-inflammatory genes 7 days after treatment. (**B** and **C**) Fluorescent images and quantitation of M1 and M2 macrophages in heart tissues, indicated by iNOS and CD206, respectively. (**D**) Representative pictures of infarcted sections stained with H&E on Day 7 after MI. Data are mean ± SD (*n *=* *3; *P <* *0.05, **P <* *0.01, ***P <* *0.001, ****P <* *0.0001, ns stands for no significant difference).

Heart tissue from mice was collected at 7 days postoperatively to evaluate the inflammation response. The mRNA levels of representative pro-inflammatory factors (TNF-α and IL-6) and anti-inflammatory factors (IL-10 and Arginase 1 [Arg1]) were analyzed using reverse transcription (RT-qPCR). As shown in Figure 5A, the pro-inflammatory factors TNF-α and IL-6 expressions were significantly elevated in the MI group compared to the Sham group. However, treatment with Exo or NAM-Exo led to a substantial downregulation of these pro-inflammatory factors while concurrently upregulating the expression of the anti-inflammatory factors IL-10 and Arg-1 in the infarcted areas. Moreover, myocardial specimens were subjected to immunofluorescence staining of iNOS (an M1 macrophage marker) and CD206 (an M2 macrophage marker) to assess macrophage presence [48]. As shown in Figure 5B and C, a significant decrease in iNOS expression and increased CD206 expression in the NAM-Exo group were observed compared to the MI and Exo groups. These findings indicate that NAM-Exo treatment effectively promotes the increase of anti-inflammatory M2 macrophages while suppressing pro-inflammatory M1 macrophages, thereby alleviating the inflammatory response. Moreover, the histological analysis using H&E staining revealed that the myocardium in the Sham group exhibited a typical structure with well-arranged cells (Figure 5D). In contrast, compared to the MI group, both the Exo and NAM-Exo groups exhibited reduced infiltration of inflammatory cells and loose fibrous tissue between myocardial cells, with the NAM-Exo group showing more pronounced effects. This reduction suggests a mitigation of the inflammatory response in the infarct area. These findings collectively indicate that NAM-Exo has the potential to alleviate inflammation, thereby facilitating better cardiac repair. This effect may be attributed to modifying exosomes with the membrane of native neutrophil-derived ABs, which enhances their targeting and retention in the injured heart and improves their efficacy in regulating inflammation [13]. In addition, the capacity of NAM-Exo to mitigate myocardial cell apoptosis following MI was evaluated using terminal deoxynucleotidyl transferase dUTP nick end labeling (TUNEL) staining assay. The NAM-Exo administration significantly diminished the number of apoptotic cells at 7 days post-injury (Figure S6), suggesting that NAM-Exo confers a more pronounced protective effect compared to untreated controls or Exo alone. This enhanced protection may be attributed to the synergistic actions of increased secretion of anti-inflammation factors and the intrinsic anti-apoptotic properties of the exosomes themselves [49].

### *In vivo* assessment of angiogenesis, cardiac fibrosis and heart function

To evaluate the therapeutic effects of NAM-Exo *in vivo*, NAM-Exo was administered via the tail vein of mice 1 day after MI and subsequently injected once a week for 4 weeks. The results were compared among the Sham (mice in this group underwent thoracotomy without LAD ligation), MI (mice in this group underwent LAD ligation and received PBS injection only), Exo (mice in this group underwent LAD ligation and received exosome injection) and NAM-Exo groups (mice in this group underwent LAD ligation and received NAM-Exo injection).

**Figure 6.**
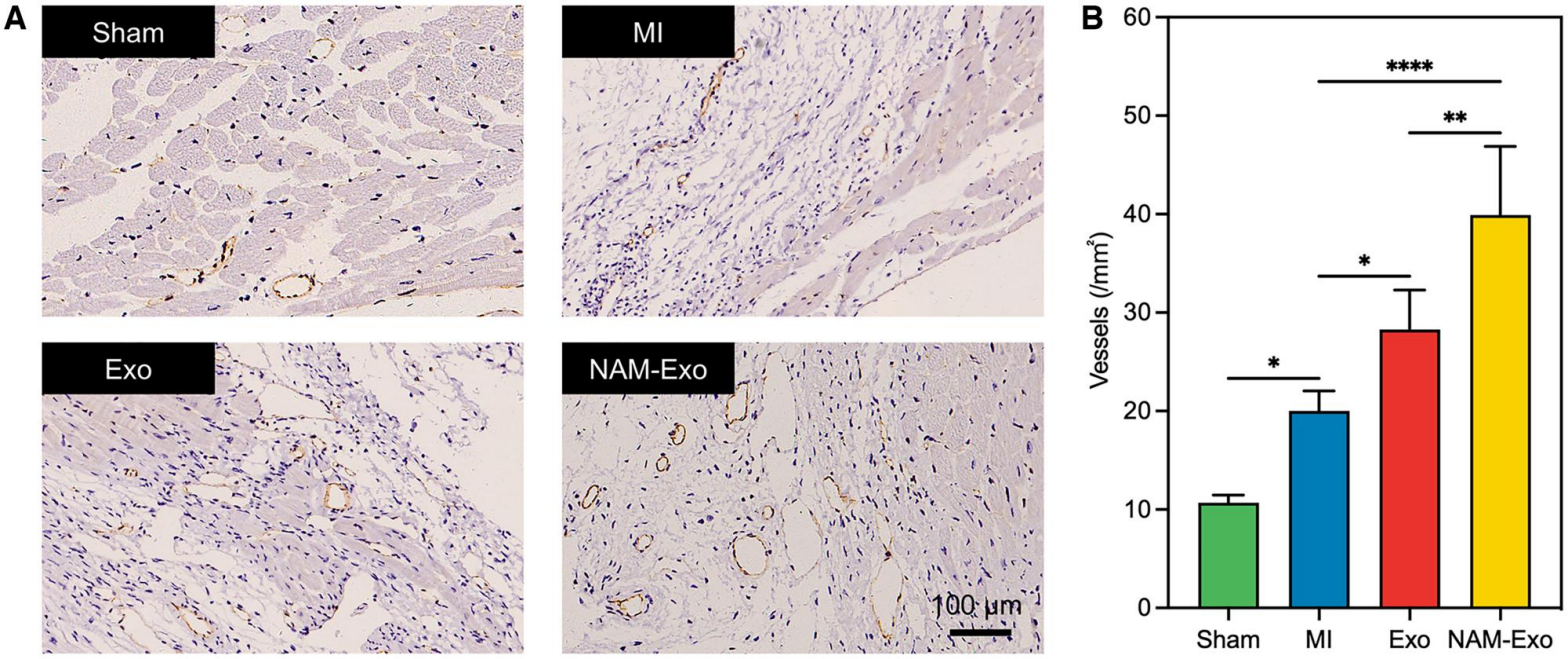
NAM-Exo therapy enhances angiogenesis 28 days after MI. (**A**) Representative images of CD31 immunohistochemical staining on heart sections. Scale bar = 100 μm. (**B**) Quantitation of vessels/mm^2^ in hearts. Data are mean ± SD (*n *=* *3; *P <* *0.05, **P <* *0.01, ****P <* *0.0001).

Post-MI healing comprises inflammation, proliferation and maturation/remodeling phases [50]. Neovascularization primarily occurs during the proliferation phase to enhance blood supply, delivering oxygen and nutrients to the infarcted site. Therefore, promoting angiogenesis benefits cardiac repair and functional recovery [51–53]. Exo and NAM-Exo were administered to MI mice to investigate neovascularization and tissues from the infarct sites were harvested 28 days post-MI. Immunohistochemical staining was performed to evaluate CD31 expression (a specific endothelial cell marker) in the harvested tissues, with CD31-positive structures indicating blood vessels. As shown in Figure 6, compared to the MI group, all groups treated with Exo or NAM-Exo exhibited significantly increased blood vessel formation. Particularly, the NAM-Exo group demonstrated the highest number of blood vessels, suggesting that NAM enhanced the pro-angiogenic potential of exosomes and improved the MI microenvironment.

**Figure 7.**
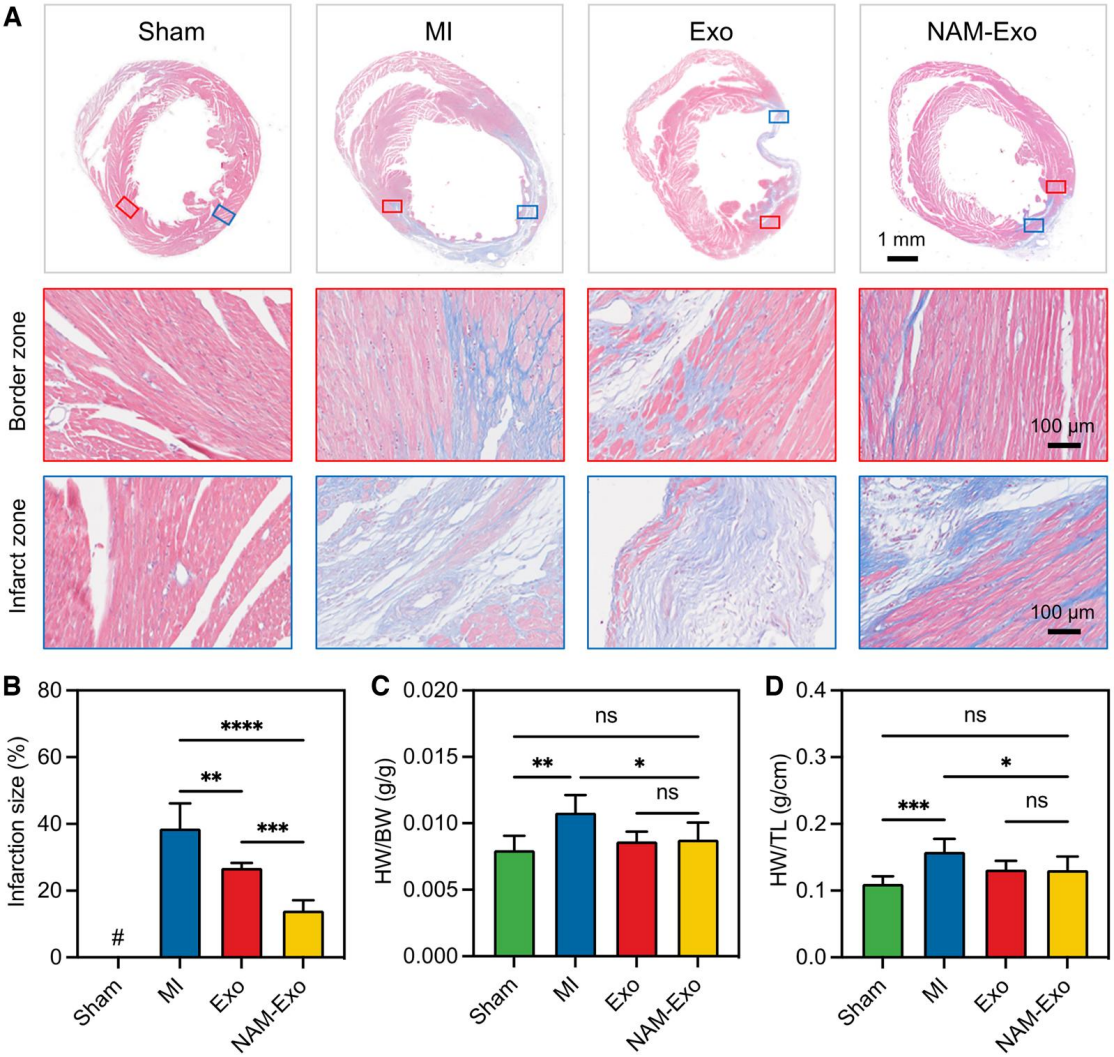
Effects of NAM-Exo on cardiac structure days 28 days post-MI. (**A**) Representative images of Masson staining on heart sections from mice treated with PBS, Exo or NAM-Exo. The panels below depict high-magnification views of the corresponding representative images of the border zone and the infarct zone. (**B**) Quantitative analysis of the infarction size is expressed as the ratio of the infarct area to the total left ventricular area. (**C**) HW/BW and (**D**) HW/TL as determined 28 days post-MI and treatment. Data are mean ± SD (*n *=* *5; *P <* *0.05, **P <* *0.01, ***P <* *0.001, ****P <* *0.0001, ns stands for no significant difference).

In the pathological remodeling process following acute MI, the healthy myocardium is gradually replaced by collagenous scar tissue, which significantly contributes to post-ischemic cardiac dysfunction and heart failure [54, 55]. Therefore, reducing myocardial fibrosis emerges as a crucial aspect of cardiac recovery. This study investigated the effect of Exo or NAM-Exo on fibrosis by performing Masson’s trichrome staining on heart sections 28 days post-treatment. Remarkably, a significant decrease in the fibrotic area within the infarct zone was observed in the NAM-Exo group compared to the Exo and MI groups (Figure 7A and B, 14 ± 0.03% vs. 27 ± 0.02% vs. 38 ± 0.07%, NAM-Exo vs. Exo vs. MI). This finding suggests that NAM-Exo treatment can reduce MI size and alleviate myocardial fibrosis, potentially delaying ventricular remodeling and facilitating cardiac repair.

Furthermore, the ratios of HW/BW and HW/TL serve as important indicators of ventricular remodeling. On the 28th postoperative day, our results revealed a significant decrease in HW/BW (Figure 7C) and HW/TL (Figure 7D) in the Exo and NAM-Exo groups compared to the MI group, corroborating previous reports on the alleviation of ventricular remodeling with exosome treatment [56].

**Figure 8.**
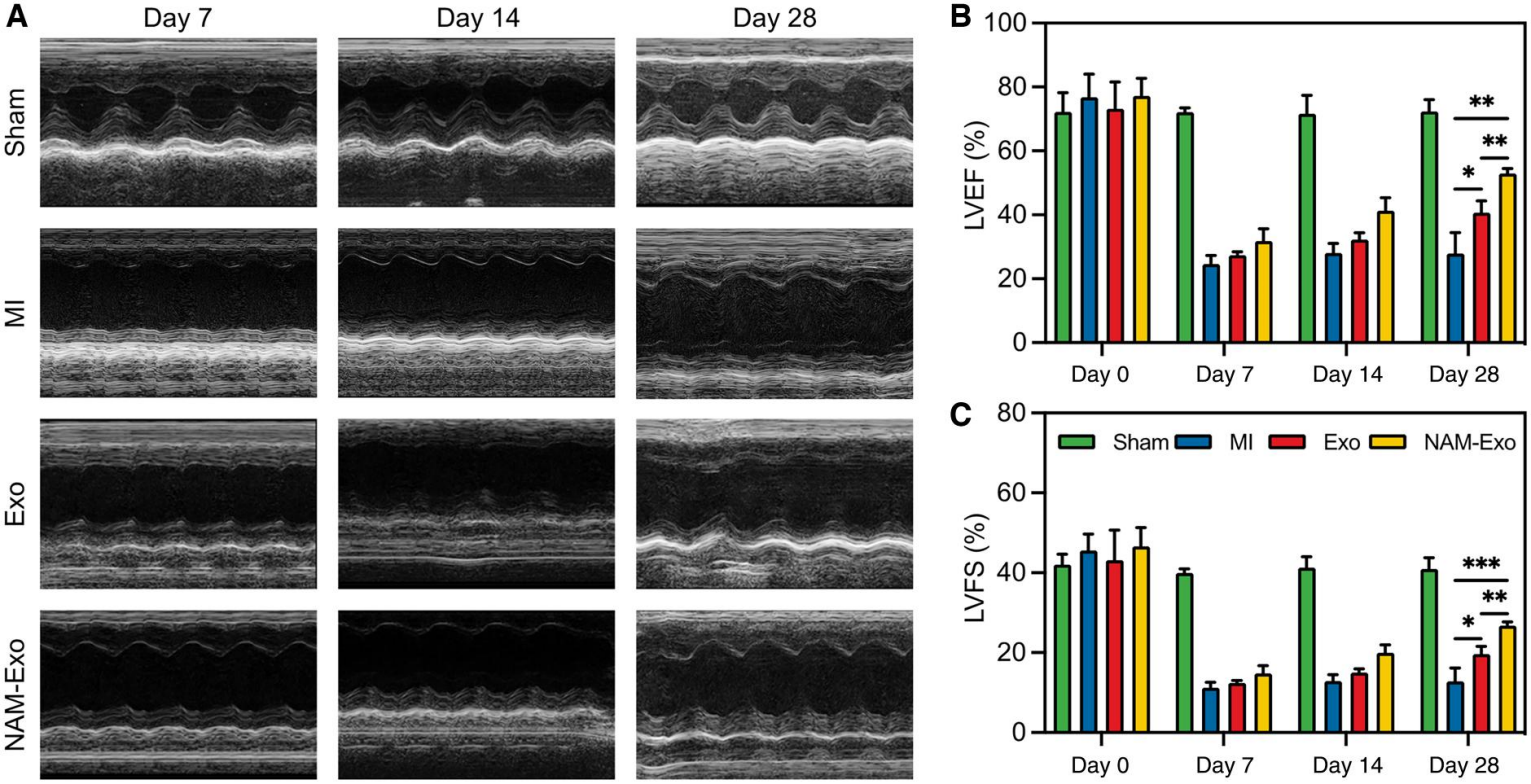
Impact of NAM-Exo on cardiac function assessed via echocardiography. (**A**) Illustrative echocardiographic images depicting various groups on Days 7, 14 and 28. Quantitative analysis of (**B**) left ventricular ejection fraction (LVEF) and (**C**) fraction shortening (LVFS) at the designated time intervals. Data are mean ± SD (*n *=* *5; *P <* *0.05, **P <* *0.01, ***P <* *0.001).

Cardiac function was assessed using echocardiography, with LVEF and shortening fraction (LVFS) being the primary evaluation indices [57]. Echocardiography studies were conducted at 7, 14 and 28-day intervals (Figure 8A and B) following treatment. In the Sham group, LVEF and LVFS measured 72.44 ± 3.30% and 40.55 ± 2.69%, respectively, at 28 days post-MI, closely resembling the cardiac function of normal mice, as documented by previous studies [58, 59]. Conversely, LVEF declined to 28.80 ± 5.67% in the MI group. However, following Exo or NAM-Exo administration, LVEF significantly increased to 40.90 ± 3.34% and 52.95 ± 1.52%, respectively, with the highest LVEF observed in the NAM-Exo group. LVFS exhibited a similar trend (Figure 8C), with values of 13.34 ± 2.83%, 19.71 ± 1.77% and 26.77 ± 0.95% for the MI, NAM and NAM-Exo groups, respectively. These results suggest enhanced contraction of the left ventricular anterior wall and partial recovery of cardiac systolic function.

Overall, our *in vivo* experiments demonstrated that NAM-Exo enhanced cardiac function by promoting angiogenesis, reducing the infarct size and inhibiting fibrosis at the infarct site. This enhancement likely stems from surface modifications of exosomes with NAM, augmenting their residence within the heart and fully harnessing their therapeutic potential. In addition, these effects may be associated with the uptake of NAM-Exo by macrophages and their subsequent anti-inflammatory transition, fostering a stable microenvironment conducive to regeneration.

### Biosafety assessment of NAM-Exo therapy

**Figure 9.**
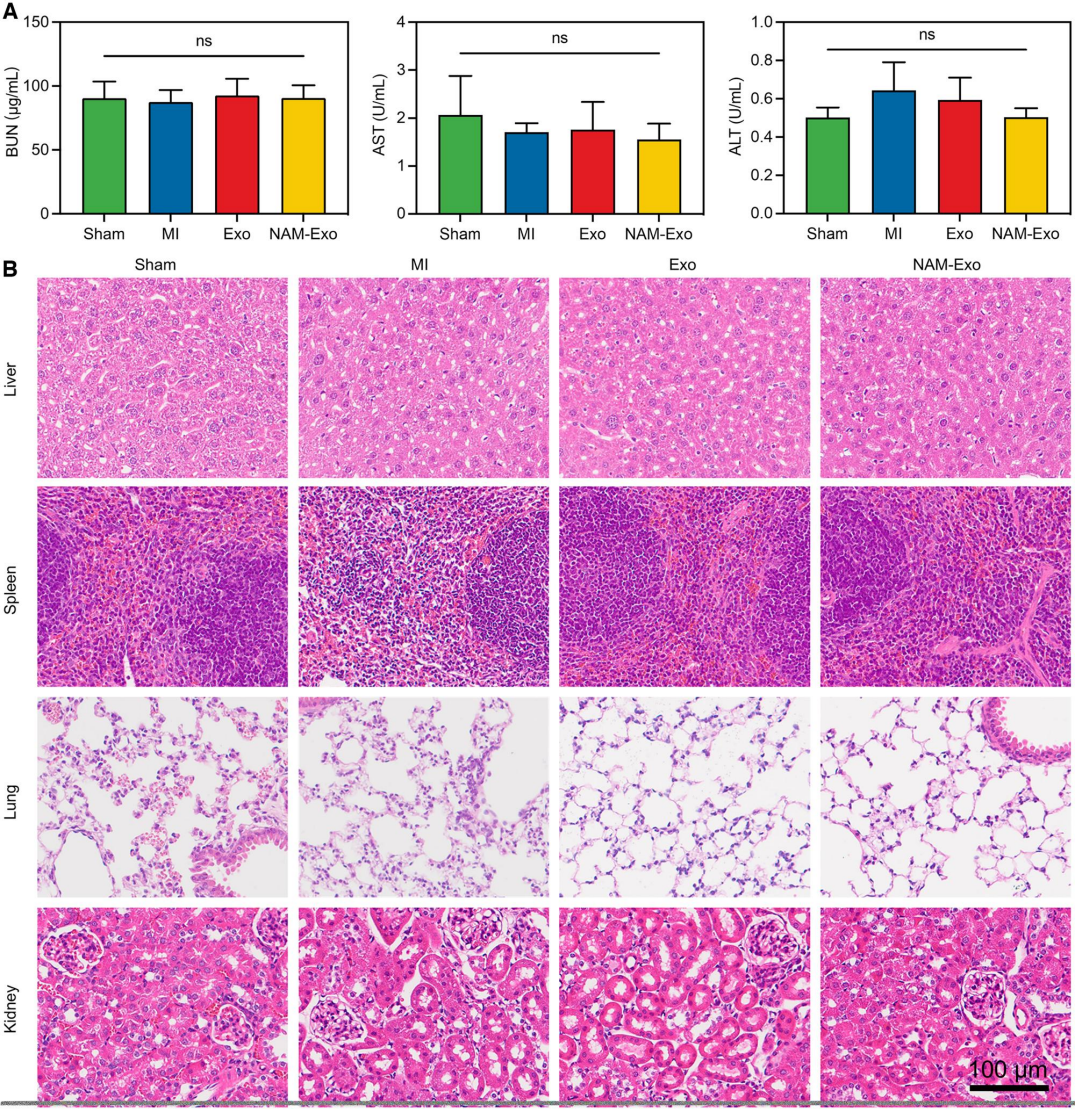
Safety verification of NAM-Exo. (**A**) Quantitative analysis of blood biochemistry, reflecting liver and kidney function from peripheral blood samples. (**B**) Hematoxylin and eosin (H&E) staining of vital organs (liver, spleen, lung and kidney) from MI mice with and without exosome treatment, demonstrating no discernible histological changes following different interventions. Data are mean ± SD (*n *=* *5, ns stands for no significant difference).

Exosomes are widely utilized in biomedicine for their safety, stability and scalability for mass production. Clinical endeavors have corroborated their *in vivo* safety and efficacy. This study scrutinized the biocompatibility and immunogenicity of NAM-Exo after systemic injection to assess their translational potential. Comparative analysis with the Sham group revealed no significant differences in hematological parameters such as alanine transaminase (ALT), aspartate transaminase (AST) and blood urea nitrogen (BUN) levels in the NAM-Exo group (Figure 9A), indicating that hepatic and renal functions remained unaffected by NAM-Exo treatment. Moreover, as shown in Figure 9B, H and E staining of major organs (including liver, spleen, lung and kidney) demonstrated no pathological alterations in tissue structure, cellular morphology or immune cell infiltration at 28 days post-treatment with NAM-Exo. These findings collectively suggest the excellent *in vivo* biosafety profile of NAM-Exo, indicating no potential organ toxicity, which is essential for its prospective clinical utilization.

## Conclusions

In summary, this study successfully augmented the adhesion of exosomes to injured endothelium and prolonged their retention time in infarcted myocardium by fusing them with AB membranes. Intravenous administration of NAM-Exo mitigated inflammation, promoted angiogenesis and enhanced the protective effect of exosomes on cardiac remodeling post-MI. Therefore, cell mimic-targeted exosomes represent a promising platform for advancing stem cell exosome-based heart repair or regeneration therapies.

## Funding

This work was supported by the National Natural Science Foundation of China (92168203), the Key Research & Development Plan of Jiangsu Province (BE2023711), the Natural Science Foundation of Jiangsu Province (BK20231314), the National Key R&D Program of China (2022YFA1104300), the Suzhou Science and Technology Plan Project (SKY2023003) and the Jiangsu Cardiovascular Medicine Innovation Center (CXZX202210).

## Supplementary data

Supplementary data are available at *Regenerative Biomaterials* online.

*Conflicts of interest statement*: The authors declare that they have no known competing financial interests or personal relationships that could have appeared to influence the work reported in this article.

## Supplementary Material

rbae145_Supplementary_Data
