# Supplementary material for: Neutrophil-derived apoptotic body membranes-fused exosomes targeting treatment for myocardial infarction
Source: Regen Biomater. 2024 Dec 14;12:rbae145. doi: 10.1093/rb/rbae145 (PMC11757162; doi:10.1093/rb/rbae145)
Supplement: rbae145_Supplementary_Data [file rbae145_supplementary_data.pdf]

Support Information

Neutrophil-Derived Apoptotic Body Membranes-Fused Exosomes Targeting Treatment for Myocardial Infarction

Jingjing Wang[a], Jingjing Li[a], Gang Su[a], Youbin Zhang[a], Zhu Wang[a], Yujuan Jia[a], Qian Yu[b,*], Zhenya Shen[a,*] Yanxia Zhang[a,*], Yunsheng Yu[a,*]

[a] Department of Cardiovascular Surgery of the First Affiliated Hospital & Institute for Cardiovascular Science, Suzhou Medical College of Soochow University, Soochow University, Suzhou 215006, PR China

[b] State and Local Joint Engineering Laboratory for Novel Functional Polymeric Materials, College of Chemistry, Chemical Engineering and Materials Science, Soochow University, Suzhou 215123, P. R. China

Corresponding Authors

*E-mail: yuqian@suda.edu.cn (Q.Y.); uuzyshen@aliyun.com (Z. S.);
zhangyanxia@suda.edu.cn (Y.Z.); yys700827@sina.com (Y. Y.)

S1 Supporting experiments

S1.1 Preparation of apoptotic neutrophils

Neutrophils were collected from fresh human peripheral blood of five healthy volunteers (23-27 years old) by using a human peripheral blood neutrophil isolation kit (Solarbio, China) according to the manufacturer's instructions. Briefly, the peripheral blood was added into buffer A and buffer C in sequence, followed by centrifugation at 850 g for 25 min at room temperature. The neutrophils were obtained by collecting the solution between the interface of buffer A and buffer C, and were then washed with phosphate-buffered saline (PBS). Neutrophils were further treated with staurosporine (Merck, Germany) at 0.5 μM for 3 h to induce apoptosis in vitro.

S1.2 Characterization of BMSCs and BMSC-derived Exosomes

BMSCs were purchased from Cell Bank and cultured in DME/F-12 complete medium at 37°C with 5% CO_2. For BMSCs verification, cells were trypsinized and washed with phosphate buffered saline (PBS). Antibodies (CD29, CD44, CD31, CD45) were incubated with BMSCs for 30 min at 4°C. After washing and resuspension with PBS, BMSCs were analyzed by a flow cytometer (Guava®easyCyte™ 8, Germany) and FlowJo software was used to investigate the surface molecular markers. Osteogenic, chondrogenic and adipogenic differentiation were conducted using the commercial kit (OriCell, China), and further determined by Alizarin Red, Alcian Blue and Oil Red O staining, respectively, according to the manufacturer's instructions.

BMSCs were cultured to 80% confluency, at which point, using DME/F-12 basic medium (Gibco, USA) containing 10% exosome-depleted FBS and 1% penicillin–

streptomycin (Gibco, USA) to culture for further 48 h. Then, the collecting medium were centrifuged at 300 g for 10 min, 2000 g for 10 min and 10000 g for 30 min to remove cells and cell debris. After filtration through a 0.22-μm filter (Merck Millipore, Germany) to further remove debris, the supernatant was ultracentrifuged at 110,000 g for 2 h to obtain exosomes, and the pellets were resuspended in PBS and stored at −80 °C for further use. The exosome concentration was determined by BCA assay (Beyotime Biotech, China). The exosomal ultrastructure and size was observed via transmission electron microscopy (TEM, JEOL, Japan). The size distribution and zeta potential were measured by a Zetasizer (Malvern, UK). Then, the expression of exosomal marker proteins (CD9, CD63, TSG101) was determined by Western blot.

S1.3 Establishment of MI model

C57B/L mice (6-8 weeks, 20 ± 2 g) were subjected to permanent ligation of the left anterior descending (LAD) branch to induce MI. Briefly, the mice were deprived of water and fasting for 12 hours before surgery. Then mice were anesthetized by intraperitoneal injection of pentobarbital (60 mg/kg) and ventilated with a rodent respirator ventilator. Following the incision of the mouse skin along the left margin of the sternum, the pectoralis major and minor muscles were carefully separated to reveal the thoracic cavity. A small cotton ball was positioned within the left thoracic cavity to shield and protect the lung lobe. The left anterior descending coronary artery (LAD) was permanently ligated using a 6-0 suture. White myocardium was observed in both the anterior wall of the left ventricle and periapical myocardium. Subsequently, closure of the chest was performed, followed by suturing the skin. MI mice were immediately

detected by electrocardiograph. The presence of pallor in the left ventricle and ST-segment elevation on the electrocardiogram was considered a successful infarct modeling.

S1.4 TUNEL staining of apoptosis

Terminal deoxynucleotidyl transferase dUTP nick end labeling (TUNEL) assays were used for tissue apoptosis. To demonstrate that NAM-Exo can improve myocardial cell apoptosis during myocardial infarction, MI mice were randomly assigned to four groups: Sham, MI, Exo, and NAM-Exo. Heart tissue was collected and frozen sectioned at 7 days post-treatment. A TUNEL apoptosis detection kit (Beyotime, China) and cTnT immunostaining were used to assay tissue cell injury and apoptosis. Samples were examined with a microscope (Olympus, Japan). All cell nuclei were stained with DAPI (blue). Apoptotic cells were dyed with TUNEL-positive nuclei (red). Cardiomyocytes were cTnT-positive cells (green).

S2 Supplementary Tables and Figures

Table S1. Sequences of the primers in RT-qPCR

**Target Gene (Mouse)**	**Primer**	**Sequence (5′–3′)**
GAPDH	Forward	AGGTCGGTGTGAACGGATTTG
	Reverse	TGTAGACCATGTAGTTGAGGTCA
IL-6	Forward	CTGCAAGAGACTTCCATCCAG
	Reverse	AGTGGTATAGACAGGTCTGTTGG
TNF-α	Forward	CCTGTAGCCCACGTCGTAG
	Reverse	GGGAGTAGACAAGGTACAACCC
IL-10	Forward	CTTACTGACTGGCATGAGGATCA
	Reverse	GCAGCTCTAGGAGCATGTGG
Arg-1	Forward	CTCCAAGCCAAAGTCCTTAGAG
	Reverse	AGGAGCTGTCATTAGGGACATC

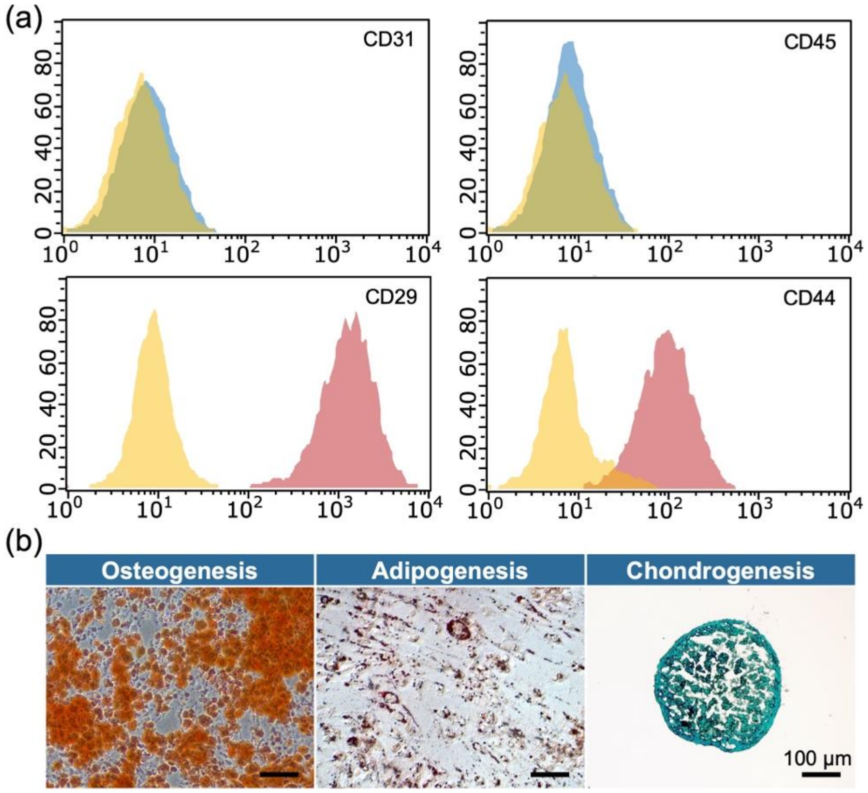

Fig. S1. Characterization of BMSCs. (a) Flow cytometry of BMSCs showed that the surface markers of CD29 and CD44 were positive, while CD31 and CD45 were negative. (b) Assessment of the trilineage differentiation capacity of BMSCs. BMSCs were successfully induced and differentiated into alizarin-red positive osteoblasts, oil red O positive lipoblasts and alicin blue positive chondrocytes using special medium. Scale bar =100 μm.

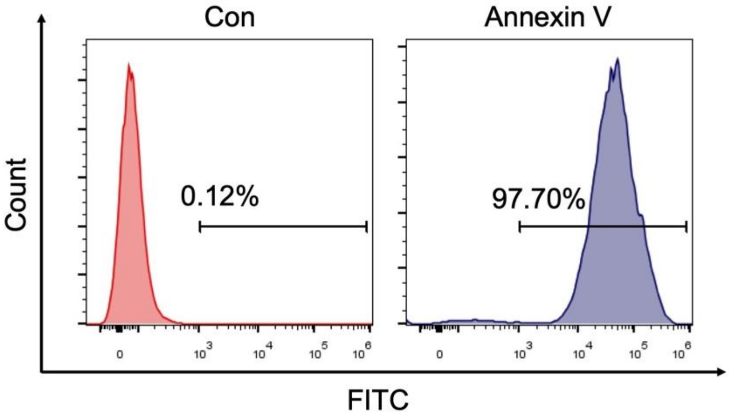

Fig. S2. The flow cytometry analysis of Annexin V-positive population in ABs.

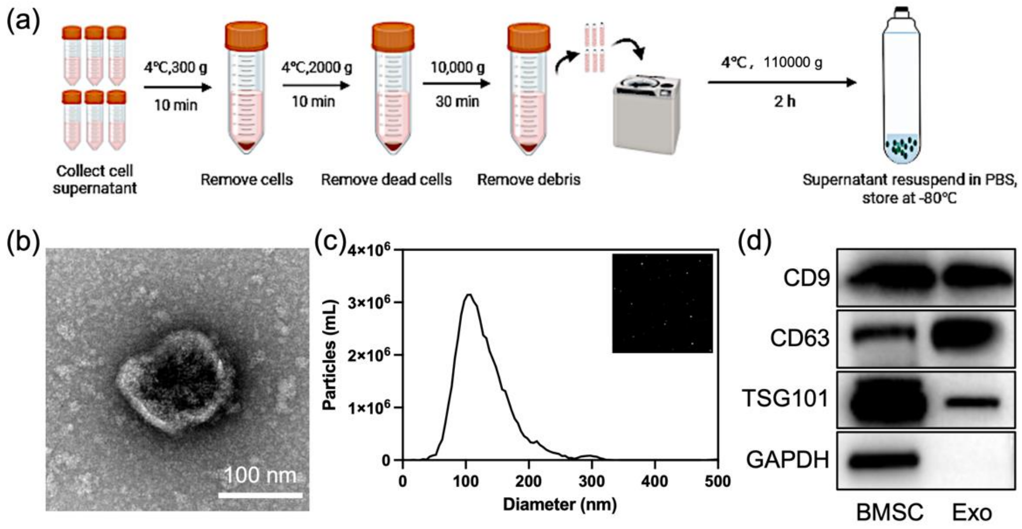

Fig. S3. Preparation and Characterization of BMSCs derived exosomes (Exo). (a) Schematic diagram of Exo preparation. (b) Representative TEM image of an Exo. (c) Representative ZetaView NTA analysis of Exo. Insert image is a screenshot from the video, showing the distribution of exosomes from the culture. (d) Western blot analysis for specific protein markers of Exo showed positive expression of CD9, CD63 and TSG101, and negative expression of GAPDH.

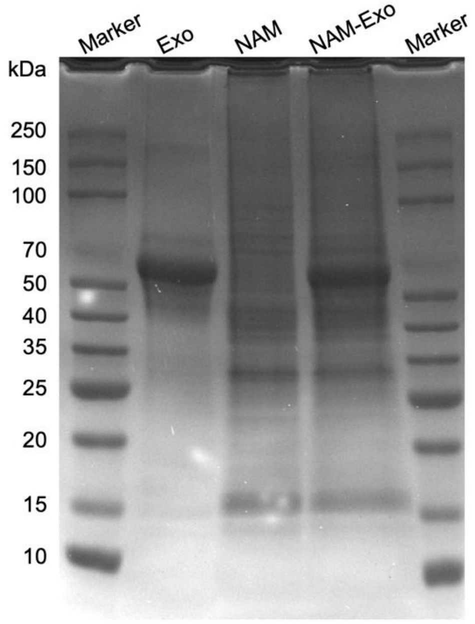

Fig. S4. Coomassie bright blue staining images of Exo, NAM and NAM-Exo.

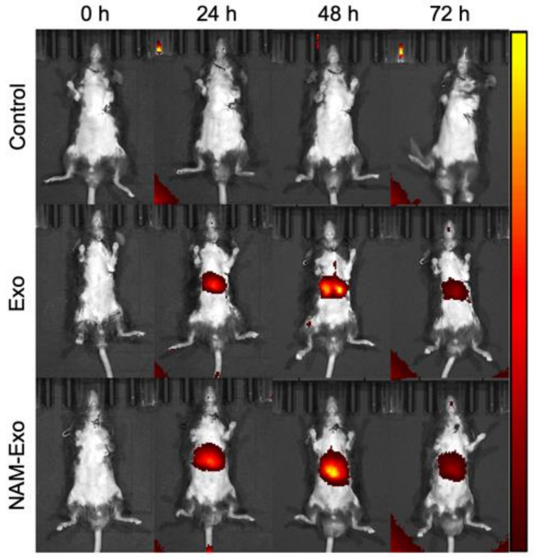

Fig. S5. In vivo images of Exo or NAM-Exo at different time points (0 h, 24 h, 48 h and 72 h).

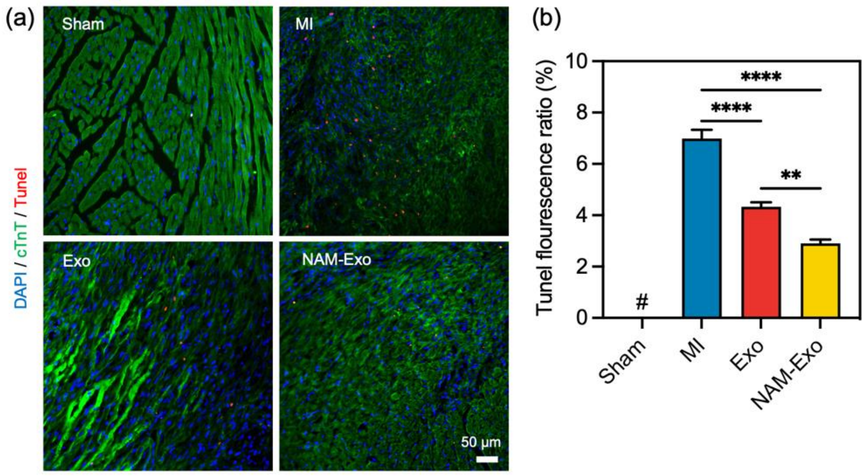

Fig. S6. (a) Representative images and (b) quantitative measurement of apoptosis in the injured myocardium as stained with TUNEL staining. Data are mean ± SD (n =3; **p < 0.01, ****p < 0.001).
